# Opuntia Ficus-Indica (OFI) Mucilage as Corrosion Inhibitor of Steel in CO_2_-Contaminated Mortar

**DOI:** 10.3390/ma14051316

**Published:** 2021-03-09

**Authors:** Andrés A. Torres-Acosta, Paola Y. González-Calderón

**Affiliations:** 1 School of Engineering and Science, Tecnologico de Monterrey, Santiago de Querétaro 76130, Mexico; 2 CH Arquitectura y Construcción, 15A Avenida Sur, Centro, Cozumel 77710, Mexico; paola_glezc@hotmail.com

**Keywords:** corrosion inhibitor, durability, half-cell potential, mortar, Opuntia ficus-indica, polarization resistance

## Abstract

The present investigation is directed to determine if a natural/botanical addition, from Opuntia ficus-indica (OFI) cactus, increases durability for cement-based materials exposed to CO_2_-laden environments (urban and industrial). The use of this botanical addition in cement-based material applications has shown good performance when these materials are exposed to chloride-laden environments, but no investigations to date have shown the performance of this addition in urban/industrial environments. Therefore, the aim of this investigation is to complement OFI mucilage performance in the most hazardous environments where most of these construction materials are naturally exposed: marine, urban, and industrial. Steel-reinforced mortar prisms, containing OFI mucilage at different addition levels (0%, 1.5%, 4%, 8%, 42%, and 95%, by water mass replacement concentration), were exposed for 14 years (5110 days) in a natural CO_2_-laden environment. Linear polarization resistance measurements were performed in a wet–dry cycle (between 5020 and 5110 days of age, after mortar fabrication) to determine the possible corrosion-inhibiting effect of OFI mucilage additions. Little corrosion-induced cracking was observed in carbonated mortars with OFI mucilage additions, compared with the carbonated control mortar that showed high corrosion-induced cracking. The electrochemical results showed corrosion-inhibiting efficiencies for steel in carbonated mortar with OFI mucilage additions of 40–70% for low OFI mucilage concentrations (1.5% and 4%), and 70–90% for medium and high OFI mucilage concentrations (8%, 42%, and 95%). Experimental findings suggest that adding OFI mucilage might be useful as a corrosion inhibitor for steel in carbonated cement-based materials (i.e., mortar) because corrosion rates and cracking initiation/propagation were decreased.

## 1. Introduction

Opuntia ficus-indica (OFI, a cactus named Nopal in Mexico) grows in semi-arid and arid environments forming thickets, regularly the size of a bush and, in some cases, as large as a 6 m tall tree. The origin of this cactus is Mexico, but this cactus is commercially grown not only in Mexico, but also in Chile, Argentina, Morocco, Italy, and parts of California, Texas, and Florida [1,2,3].

Organic additives have been used in the production of concrete and mortars for decades, improving their properties in fresh and hardened states [1,2]. For example, cactus mucilage from OFI has been used as an addition in lime mortars for restoration work on historic lime and/or adobe buildings in Latin America [3,4]. Furthermore, their use in hydraulic concrete has shown flow improvement, retarded setting times, increased workability, and increased mechanical strength at a later age [2,4,5].

Two OFI additions, for cement-based material durability improvements, were evaluated previously: OFI mucilage [1,2,3,4,5,6] and dehydrated OFI [7,8,9,10]. As explained in previous investigations, based on scanning electron microscopy–energy dispersive X-ray spectroscopy (SEM/EDS) and X-ray diffraction (XRD) characterization, OFI mucilage is a polysaccharide with several amino acids and sugars (arabinose, galactose, galacturonic acid, rhamnose, and xylose residues), as well as minerals such as calcium, potassium, and sodium [1,2,3,11]. Cladode mineral concentration varies depending on harvest time (dry or wet season) and plant age [11]. A further chemical composition of OFI mucilage, specialized biopolymer, can be found elsewhere [3,4]. These mineral contents, especially calcium, mean that OFI mucilage remains stable in highly alkaline media over long time periods, and long-term degradation of this botanical product in cement-based materials should be not possible.

This biopolymer (OFI mucilage) is produced by first cutting OFI cladodes into small cubes and placing them in water for two days at most (maceration technique). Another extraction process is to boil the OFI cladode cubes in water for a couple of hours (instead of days for the maceration extraction) [3,4,5,6].

OFI mucilage also has showed good performance as a corrosion inhibitor for steel in chloride-contaminated alkaline solution, mortar, and concrete [1,3,9,10]. This performance was apparently because this biopolymer reacts with the steel surface to form a more close-packed and thicker oxide-polymer protective layer during mortar–concrete hardening, which increases the propagation period when chlorides reach the reinforcing steel.

Results presented in previous investigations dealt with the corrosion inhibition of dehydrated OFI and OFI mucilage, for steel in cement-based materials (mortar and concrete) exposed to chloride-laden environments [1,3,9,10]. However, the corrosion inhibition properties of the same OFI mucilage additions in CO_2_-laden environments (i.e., urban or industrial) have not been evaluated yet. Therefore, the present study objective was to determine the electrochemical performance of carbon steel embedded in cement-based mortars containing the same OFI mucilage proportions as in the previous investigation [3], in long-term (5110 days or 14 years) CO_2_-laden natural environment exposure, and to define if this natural/botanical addition may be suitable for durability enhancement in other hazardous environments (urban and industrial).

## 2. Materials and Methods

### 2.1. Materials

OFI mucilage preparation in this investigation was from boiling OFI cladodes in water (previously sliced into small cubes, approximately 2 cm × 2 cm × 2 cm) for 45–50 min. The water:cladode ratio was 1:1 by mass. The final product (slime and cladode cubes) was filtered to obtain just the slime. Slime was kept inside glass containers to avoid decomposition, before mortar mixture fabrication (OFI slime was obtained one day prior to mortar mixing). The cement used for this investigation was CPO cement (Mexican designation for ASTM Type I Portland cement) [12]. A nominal 1:3 cement:sand proportion was used for mortar fabrication, using silica sand as fine aggregate for these mortar mixtures. Water (w) or water + mucilage (w + m) content was constant for all mixtures: 650 mL per 500 g.

A total of six mixtures were fabricated that used the same amount of cement and sand, varying only the amount of OFI mucilage added to the mixing water. Even though the w or w + m content was constant, plastic consistency was determined using the ASTM C-230 Standard [13] to determine possible changes in mortar fluidity due to OFI mucilage concentration in the mortar. Cube fabrication was performed later using the ASTM C-305 [14] Standard.

The OFI mucilage additions were 0% (control), 1.5%, 4%, 8%, 42%, and 95% by water replacement. Mixture proportions and flow characteristics for each one of the mortar treatments used in this and previous investigations are shown in Table 1 [3].

### 2.2. Specimen Geometry

Reinforced prisms (2 cm × 5.5 cm × 8 cm) were used to perform the electrochemical characterization of the tested mortars. Prisms were reinforced with two 6 mm diameter smooth bars (6.7 cm long, ~13 cm^2^ area in contact with mortar). The specimens also included a 6 mm diameter carbon bar internal reference electrode (IRE) to perform electrochemical tests [3].

Prisms were cured in a high-humidity chamber (~95% relative humidity, RH) for a period of 21 days after casting. Finally, prisms were retrieved from the high-humidity chamber and placed in an office complex, at room temperature and relative humidity (22 ± 3 °C and 60 ± 5% RH), until experimentation started. Electrochemical experimentation was initiated 5020 days after fabrication and ended ~5120 days after mortar fabrication. Results obtained from early dates were presented in previous investigations [3,4,5].

### 2.3. Electrochemical Characterization

The corrosion-inhibiting effects of such natural additions on steel rebar, in a long exposure period in an urban-like environment, were evaluated from linear polarization resistance (LPR) measurements. To determine the possible electrochemical performance of this natural addition as a function of mortar’s water absorption, a dry–wet cycle was performed (between 5020 and 5110 days of specimen age). This investigation shows results obtained within this time stage only, even though electrochemical characterization was performed periodically (at least once every six months), presented elsewhere [3,5].

Apparent polarization resistance (R_P_) values of the steel rebar after this long exposure period in a natural CO_2_-laden environment, and during this single dry–wet cycle, were performed using a Gamry Reference 600 potentiostat. A three-electrode configuration was used to perform this LPR technique, by using the two smooth steel bars (one as a counter and the other as a working electrode) and a carbon rod as the internal reference electrode (IRE).

During LPR testing, half-cell potential (HCP) was recorded between the embedded steel and the carbon IRE before the potentiostat started polarizing the steel smooth bar. Once the LPR technique ended, the HCP between the carbon IRE and a copper/copper sulfate reference electrode (CSE) was recorded, and the smooth steel bar potential was then converted to this CSE reference.

The LPR measurement setup was described in previous investigations [3,4,5]: the impressed potential varied from 0 to +20 mV from the open circuit potential (OCP) in the cathodic direction, and a scan rate of 0.05 mV/s. The rebar R_P_ value was estimated from the slope of the final 10–20 mV portion of the potential–current scan, which was normally a straight line. After LPR testing, the electrolyte electrical resistance (Rs) was measured to determine the IR drop due to possible high resistivity of the electrolyte (mortar). A Model 400A McMiller Soil Resistance Meter was used for R_S_ determination. The R_S_ value was then subtracted from the LPR test estimation, resulting in the apparent Rp value.

A cyclic dry–wet procedure was performed in one cycle. The dry cycle HCP/R_P_ measurements were performed in the regular dry condition of the mortar prisms as a starting condition. The wet cycle lasted 7 days, and the prisms were placed in plastic containers with tap water during this cycle. The wet cycle HCP/R_P_ measurements were performed with the prisms in the water, using same three-electrode configuration. During the final dry stage, several HCP/R_P_ measurements of the reinforcing steel followed the drying process of the mortar prisms and the electrochemical performance of the steel bar in the mortar during this humidity loss.

### 2.4. Mortar Crack Survey

Prisms were examined visually for surface cracks after the electrochemical characterization. Crack widths (>0.1 mm) were measured along each crack using a crack width matching reference card. This matching card is made of acrylic, and lines of different thicknesses are impressed on its surface. The reference card is placed on top of the surface crack in the mortar prism. Then, a visual matching between the surface crack and the reference card defines the crack width and this value is recorded.

Since this is an ongoing investigation, prisms are maintained in in same CO_2_-laden environment (22 ± 3 °C and 60 ± 5% RH) for further evaluations until all prisms present corrosion-induced surface cracking.

## 3. Results

### 3.1. Half-Cell Potential Measurements

**Figure 1:**
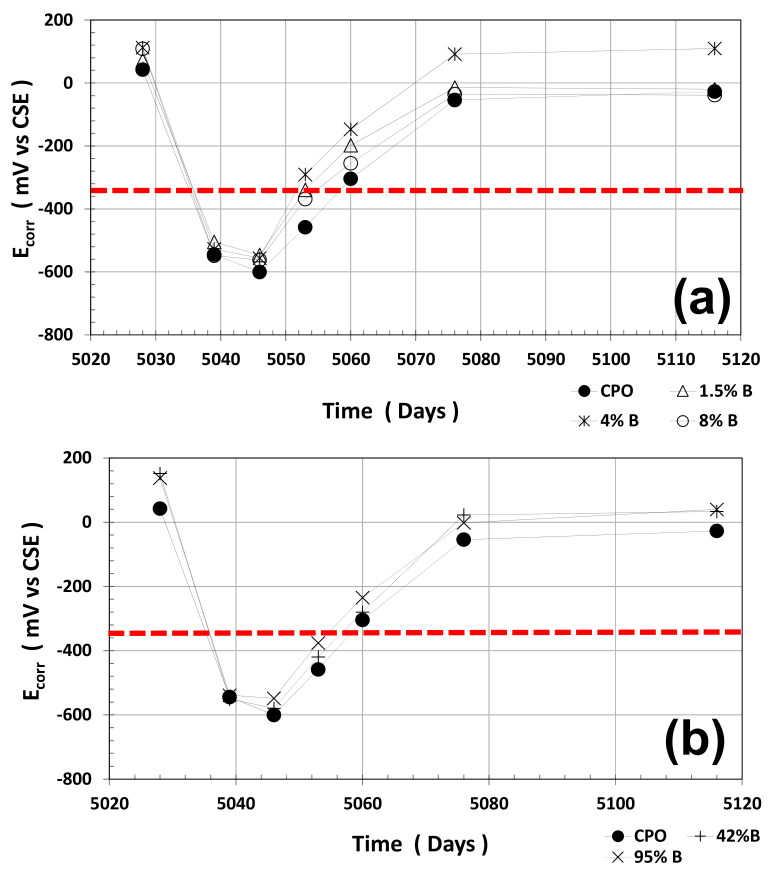
Half-cell potentials (HCPs) vs. time: (**a**) control vs. low Opuntia ficus-indica (OFI) mucilage concentration; (**b**) control vs. high OFI mucilage concentration.

Average half-cell potentials (HCPs) and average LPR measurements were performed during the time period (average of four steel 6 mm diameter bars): two dry cycle measurements and one wet cycle measurement. Average HCP measurements are shown in Figure 1.

As observed from this figure, average HCP values for steel in control mortar (OPC mortar) were always more negative than for steel in all OFI mucilage mortar treatments. Still, the performance of steel is the same in all mortar treatments: more positive HCPs when mortar is in a dry stage, and when wet, HCPs were more negative.

The red horizontal dotted line in Figure 1 at HCP = −350 mV (vs. CSE) is defined as the threshold value between passive (more positive than −350 mV) and active (more negative than −350 mV) corrosion of steel in cement-based materials.

It is clear that the average HCP values when mortar is dry present a pseudo passive state of corrosion for the embedded steel, but when water is absorbed, the average HCP drops dramatically to values where steel is considered active. After the mortar prisms were placed in the dry cycle at ambient humidity (60 ± 5% RH), steel average HCP values return to the initial range values of passive steel (~0 mV vs. CSE).

### 3.2. Rp Measurements

**Figure 2:**
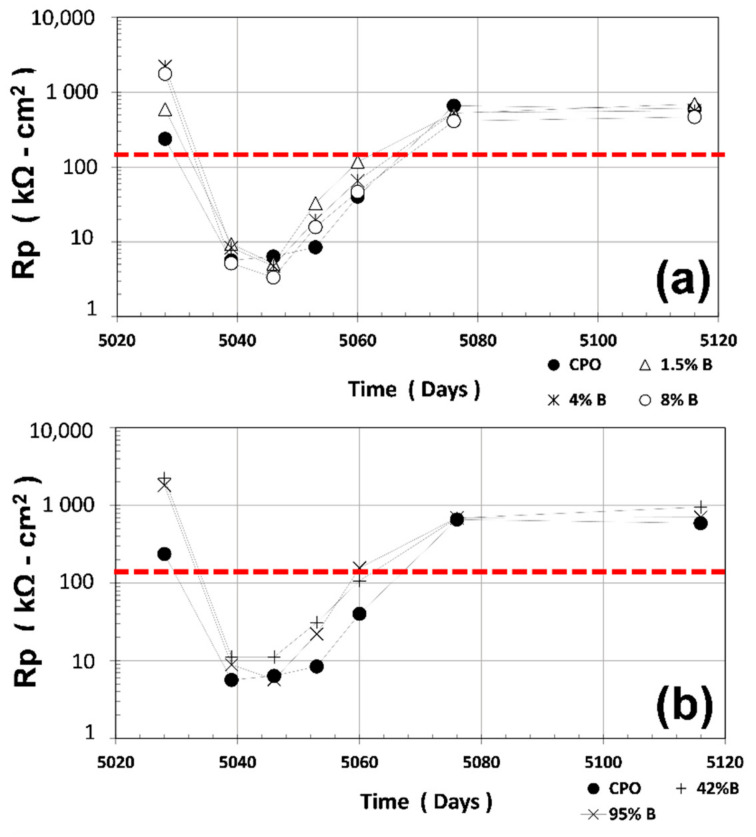
Average polarization resistance (R_P_) vs. time: (**a**) control vs. low OFI mucilage concentration; (**b**) control vs. high OFI mucilage concentration.

Average R_P_ measurements (average of four steel 6 mm diameter bars) are shown in Figure 2. The red horizontal dotted line in Figure 2 at R_P_ = 130 kΩ cm^2^ corresponds to the threshold values between passive and active corrosion of steel in cement-based materials (obtained from B = 0.026 V and corrosion rate, i_CORR_ = 0.2 μA·cm^−2^, and substituting R_P_ in the relation defined previously) [3].

As observed in Figure 2, average R_P_ values for CPO mortar (control) were smaller than the OFI mucilage mortar treatments when measured in the first dry cycle (dry for several years): 200 kΩ cm^2^ vs. 2000 kΩ cm^2^ for low OFI mucilage mortar treatments (7.5 times less). When the mortar prisms were immersed in tap water, average R_P_ values for control CPO steel bars were closer to the OFI mucilage mortar treatment steel bars.

However, when the prisms were retrieved from the water container, average R_P_ values were smaller than the OFI mucilage treatment mortars: 50% smaller than low OFI mucilage concentration mortars (1.5% B, 4% B, and 8% B), and 90% smaller than high OFI mucilage concentration mortars (42% B and 95% B). At the end, when the internal humidity of the mortar approached the initial value, average R_P_ average values for control and OFI mucilage treatments were identical.

### 3.3. Rs Measurements

**Figure 3:**
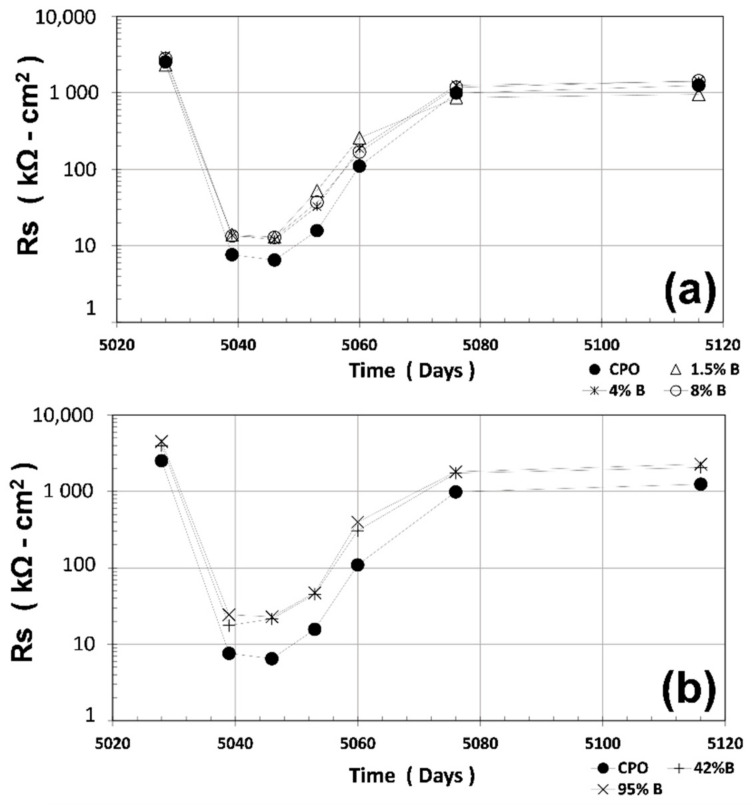
Average mortar electrical resistance (R_S_) vs. time: (**a**) control vs. low OFI mucilage concentration; (**b**) control vs. high OFI mucilage concentration.

Once the R_P_ experiments had ended, R_S_ measurements using a similar three-electrode configuration were recorded. The results of the average R_S_ are shown in Figure 3 (average of four steel 6 mm diameter bars). Again, all OFI mucilage mortar treatments had higher average R_S_ values as compared to CPO mortar (control). The difference is more noticeable when OFI mucilage concentration is high: the average R_S_ for OFI treatment mortars is 90%–160% higher than CPO mortar.

### 3.4. i_corr_ Estimates

As explained in a previous investigation, R_P_ values were used as an indirect measure of the corrosion rate of steel in the tested mortar treatments by using a well-known relationship: i_corr_~B10^6^/R_P_, where i_corr_ is the apparent corrosion rate (in μA·cm^−2^), B is a constant obtained with the Tafel slopes (in V) and equal to ~0.026 V for active steel and ~0.052 for passive steel [3].

**Figure 4:**
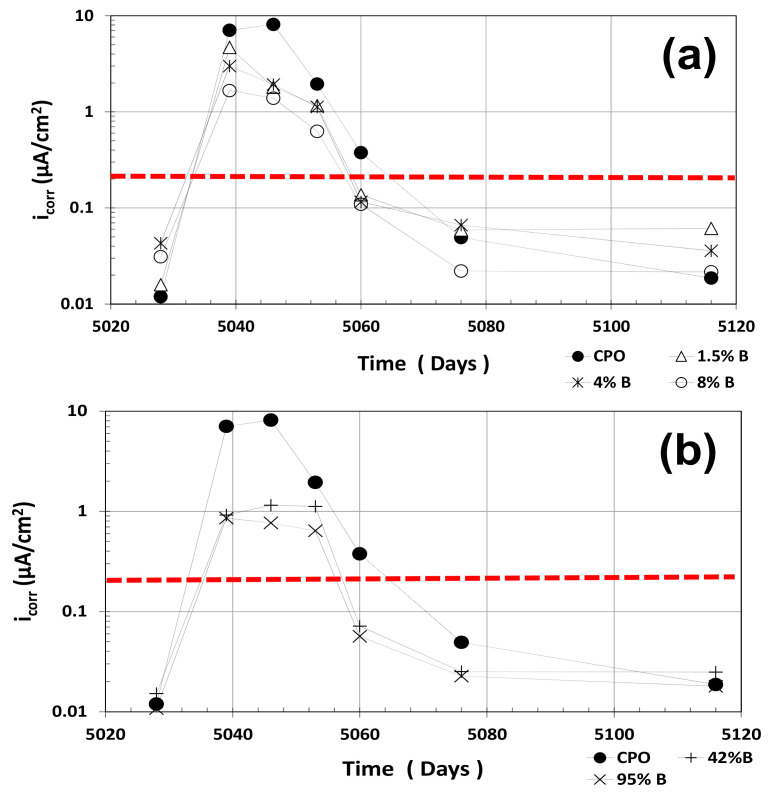
Average corrosion rate estimates (i_corr_) vs. time: (**a**) control vs. low OFI mucilage concentration; (**b**) control vs. high OFI mucilage concentration.

Figure 4 shows the average i_corr_ estimates for all mortar treatments. The red horizontal dotted line in Figure 4 at i_corr_ = 0.2 μA·cm^−2^ corresponds to the threshold values between passive and active corrosion of steel in cement-based materials. As observed in Figure 4, OPC mortar average i_corr_ values were somewhat smaller than the estimates from low OFI mucilage mortar treatments (1.5% B, 4% B, and 8% B) in the first dry cycle, but were higher than high OFI mucilage concentration treatments (42% B and 95% B).

When the mortar prisms were placed in tap water, average i_corr_ estimates for OPC (control) mortar increased to almost the 10 μA·cm^−2^ range, as compared to OFI mucilage mortar treatments, which were 1–5 μA·cm^−2^ only. Once the prisms dried after some days outside of the water containers (second dry cycle), average i_corr_ values of the low-concentration OFI mucilage treatments were twice as high as OPC mortar, but the high OFI mucilage concentration mortar treatments were similar to the control OPC mortar. However, all treatments in the second dry cycle showed very low average i_corr_ values, showing passivity in all of them.

### 3.5. Mortar Crack Survey

**Figure 5:**
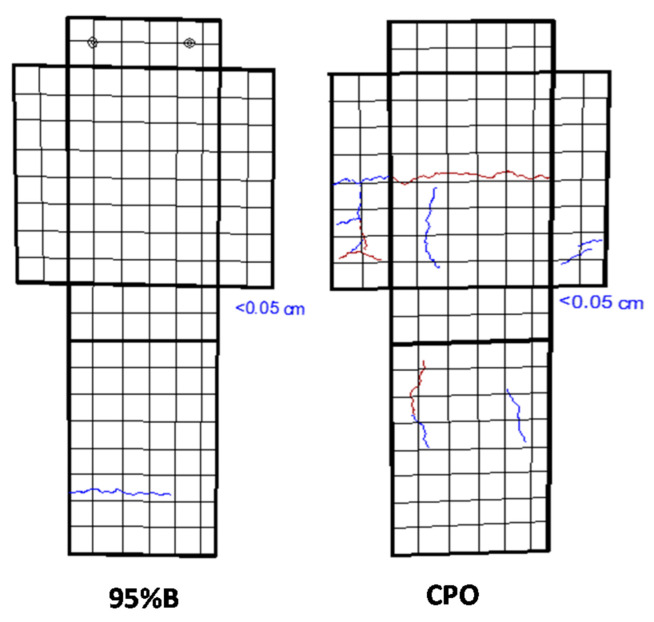
Typical surface crack survey.

After 5110 days of CO_2_-laden natural exposure inside the laboratory, some cracks were observed on the mortar prism surfaces. Figure 5 shows two of those mortar crack surveys for a 95%B mortar prism and a control CPO mortar prism. The position, length, and width of such cracks were recorded (two mortar prisms per mortar mixture, with a total of four steel bars embedded). A general trend was observed from this crack surface: higher number, length, and width of cracks were observed in the control mortar.

**Figure 6:**
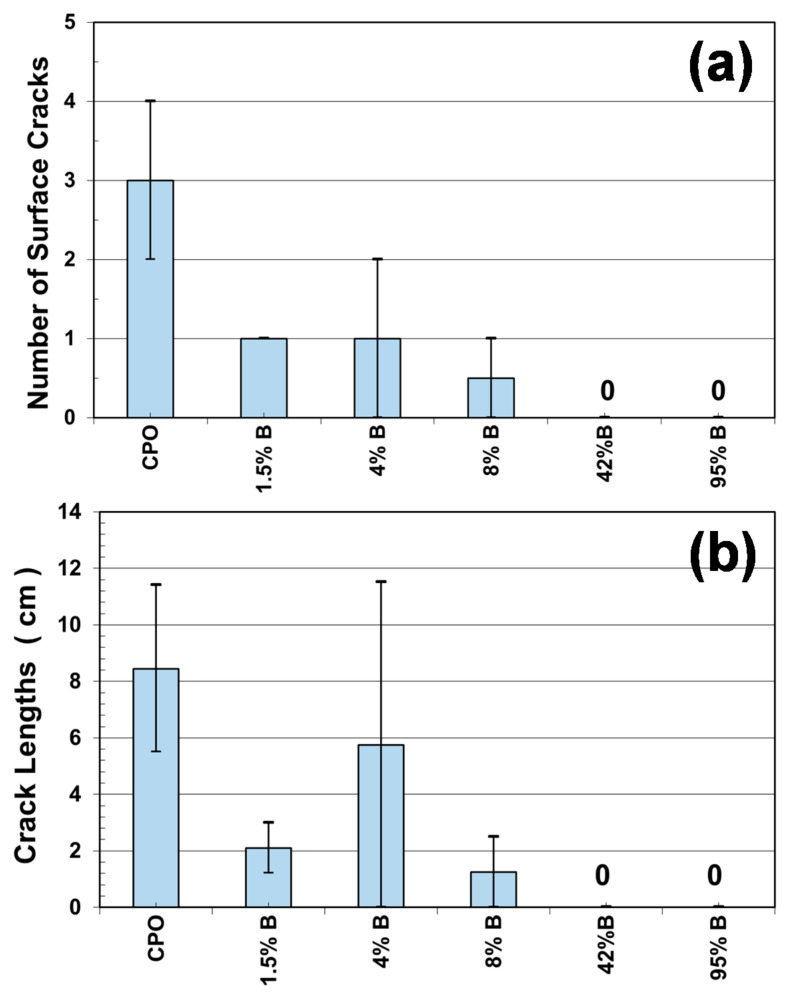
Results from the crack survey: (**a**) number of surface cracks and (**b**) crack length measurements per mortar type.

Figure 6a shows the number and total crack length measured on each of the mortar prisms evaluated, where CPO mortar presented an average of three cracks, as compared to an average of one crack for low-concentration OFI mucilage mortar (1.5% B, 4% B, and 8% B), and no cracks for high OFI mucilage concentration mortars (42% B and 95% B). As observed from Figure 6b, all embedded steel bars (four per mixture) in the control mortar (CPO) generated corrosion cracks, and the total length of cracks in the two control mortar prisms were between 6 cm and 12 cm. For low-concentration OFI mucilage mortar prisms, there is a decrease in the value of the total length of the surface cracks, although 4%B mortar reached the same length of 12 cm as in control mortar in one of the prisms, but in the others, no corrosion cracking was observed. Finally, high-concentration OFI mucilage mortar prisms did not present surface cracks in the four prisms (two prisms per mortar mixture).

These crack appearance observations helped us to visualize a possible trend where OFI mucilage addition increased the time before corrosion propagation, or the time before the appearance of corrosion surface cracks. A similar performance was observed in a previous investigation with chloride exposure, where OFI mucilage mortar prisms did not show any surface cracks after 1000 days of chloride exposure by wet–dry ponding cycles [3].

### 3.6. Inhibitor Efficiency

Average inhibition efficiency (IE in %) estimates were calculated from Equation (1) [3,9]:

(1) $$\mathrm{IE}\%=\frac{R_{t0}^{-1}-R_{ti}^{-1}}{R_{t0}^{-1}}\times 100$$

where R_ti_ and R_t0_ are the apparent polarization resistances with and without OFI mucilage additions, respectively, at time t.

**Figure 7:**
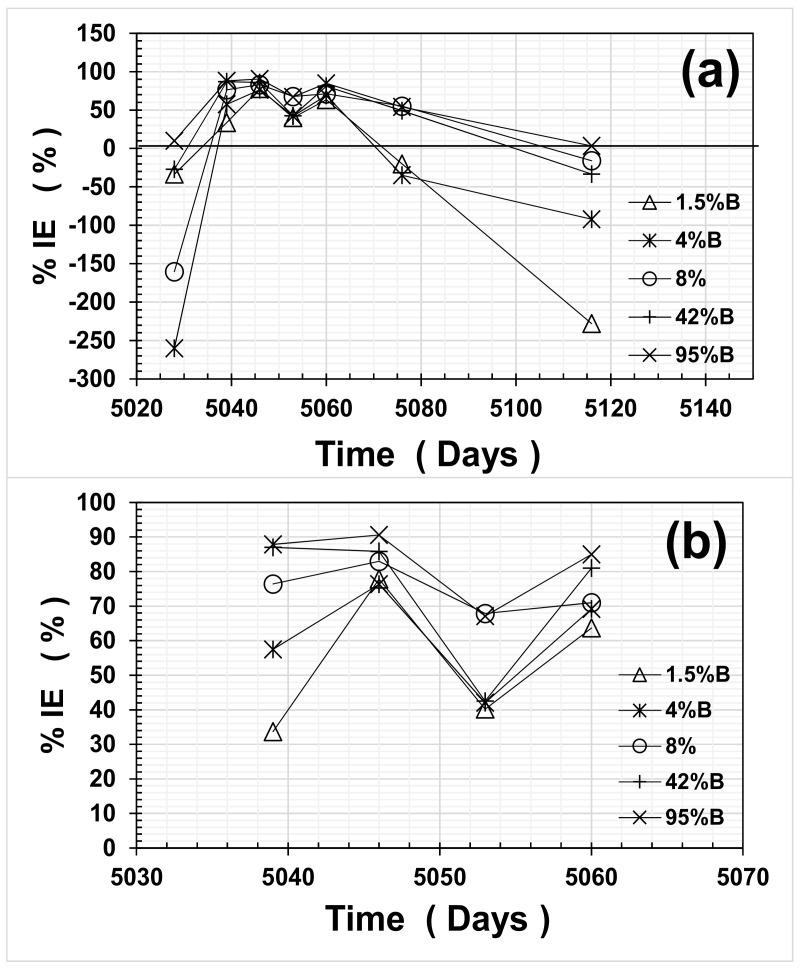
Inhibitor efficiency (%IE) vs. time: (**a**) entire experimental time; (**b**) during wet cycle.

Figure 7 presents the results of average IE% as a function of time, for the entire experimental period (Figure 7a) and the wet cycle period only (Figure 7b). As observed in Figure 7, the average IE% values for all OFI mucilage mortars were quite variable, and presented negative values when the mortar was in both dry cycles. This performance could be due to the fact that OFI mucilage retains water inside the mortar, as observed from previous investigations regarding the water absorption/desorption of mortars containing this biopolymer [3,4,5,6,7,8]. Nevertheless, i_corr_ ranges in the dry cycle were very small, similar to passive steel in cement-based materials (between 0.01 and 0.06 μA·cm^−2^), thus no detrimental effects could be found in this dry environment for such low average i_corr_ values.

On the other hand, during the wet cycle, the average i_corr_ estimates for steel in control mortars (CPO) reached up to ~10 μA·cm^−2^ as compared to >1 μA·cm^−2^ for all mortars with OFI mucilage additions. Average inhibitor efficiencies (%IEs) during the wet cycle are shown in Figure 7b and were between 30% and 70% for 1.5%B and 4%B, respectively. For 8%B mortar, the average %IE increased marginally and values ranged between 70% and 80%. For high OFI mucilage concentration mortars, the average %IE values reached between 70% and 90%, and only one average value for 42%B reached as low as an %IE of 40%.

## 4. Discussion

Mortar cubes were used to physically characterize mortars evaluated with and without OFI mucilage additions in complementary investigations [4,5]. As explained in the previous section, the results obtained with the mortar cubes in reference [4] included the following determinations: the saturated electrical resistivity (ρ), the percentage of total void content, capillary absorption and effective porosity, compressive strength, and the carbonation front. From all of these physical parameters listed, the most important for the present study was if the mortar was carbonated, in other words, if the pH of the mortar was reduced from 13–13.5 (for non-carbonated cement-based materials) to pH values close to 9–10.

Average carbonation depths (X_CO2_) obtained in a complementary investigation [4] for CPO mortar (control) were 16 ± 2 mm, compared to OFI mucilage mortars with an average X_CO2_ of 9 ± 1 mm. Considering that both smooth steel bars in the mortar prisms had a 5 ± 1 mm mortar cover, all steel bars were surrounded by carbonated mortar, regardless of mortar treatment.

**Figure 8:**
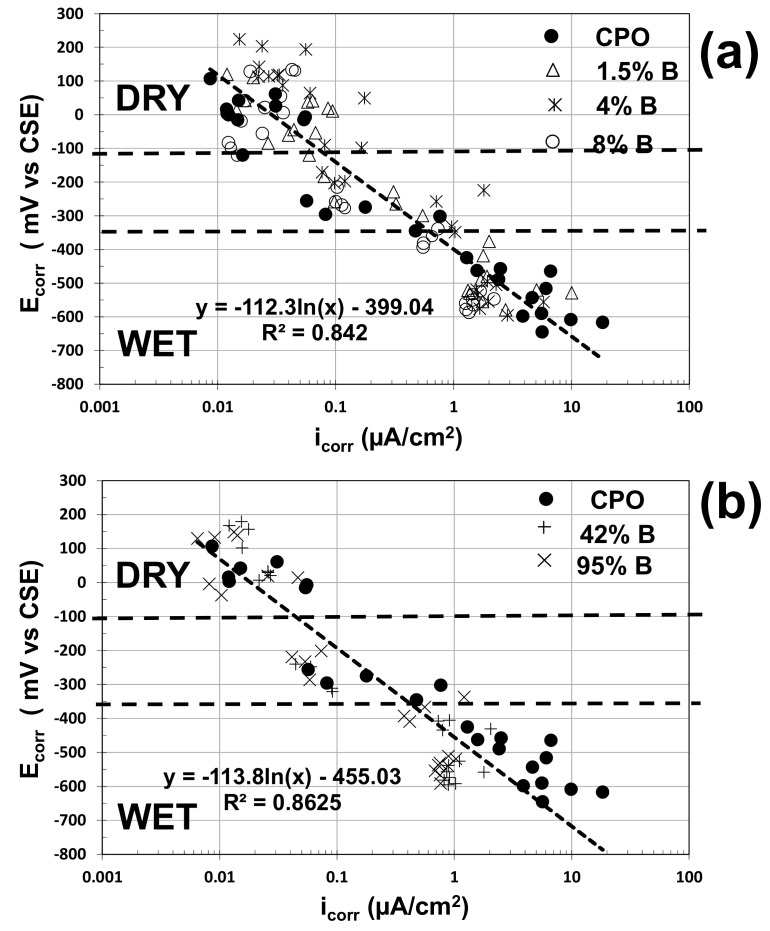
Composite plots between the electrochemical parameters E_corr_ and i_corr_ for: (**a**) control vs. low OFI mucilage concentration; (**b**) control vs. high OFI mucilage concentration.

The electrochemical performance of steel in carbonated mortar could be analyzed by plotting the HCP vs. the i_corr_ estimates for each one of the tests performed in this experimental program. Figure 8 presents this empirical correlation between HCP (now called corrosion potential, E_corr_) and i_corr_ estimates.

As observed from this composite plot, the slopes of the correlation equations were identical for low (Figure 8a) and high (Figure 8b) OFI mucilage concentrations, and almost identical to what was observed from a previous investigation on the electrochemical performance of steel in concrete columns [15]: in a semi-log scale graph, the E_corr_ vs. i_corr_ slope of steel in concrete was between 100 and 120 mV per decade.

The data were separated depending on the internal humidity in the mortar (dry, wet, and in between both zones where the mortar prisms were removed from the water containers and mortar started to dry). As observed in Figure 8, there is no electrochemical performance difference of the embedded steel in the dry zone. On the other hand, in the wet zone, there are some differences between control data and OFI mucilage data: higher i_corr_ estimates and more negative E_corr_ values for control mortar, with more marked differences for high-concentration OFI mucilage mortar.

**Figure 9:**
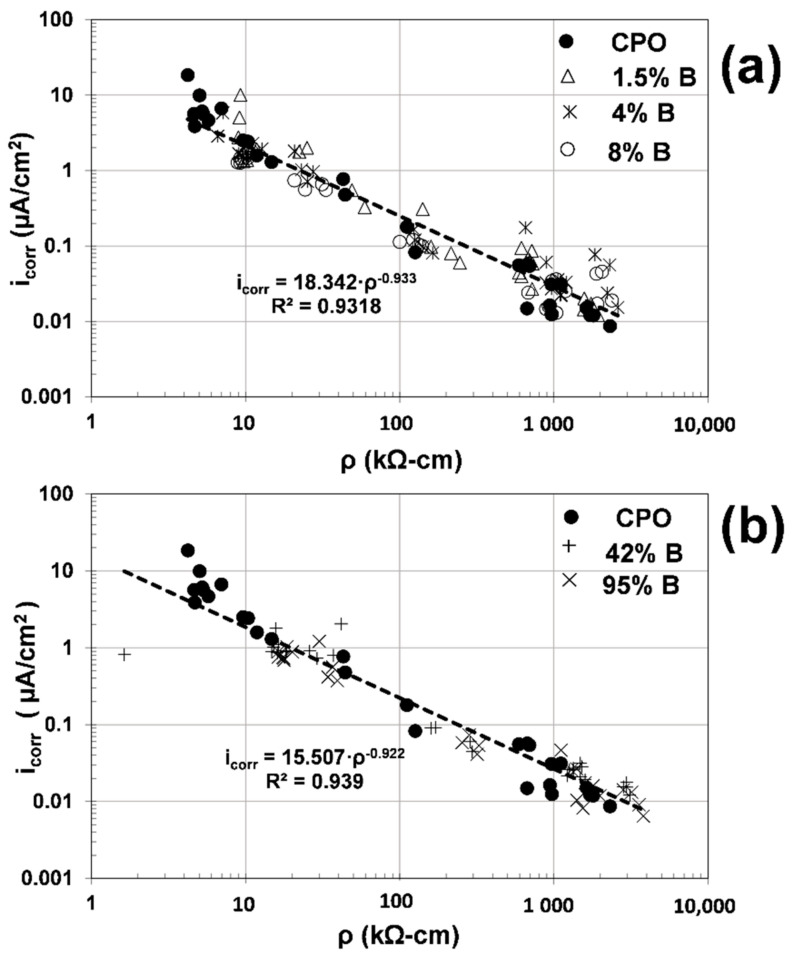
Composite plots of the electrochemical parameters of electrical resistivity of the mortar (ρ) and i_corr_ for: (**a**) control vs. low OFI mucilage concentration; (**b**) control vs. high OFI mucilage concentration.

Another important piece of information obtained with the electrochemical testing was the effect of the mortar electrical resistivity on the corrosion performance of steel in this carbonated mortar medium. In each LPR test, R_p_ and Rs were recorded and used to estimate i_corr_ of the steel bar and the electrical resistivity (ρ) of the mortar, respectively. Previously, it was explained how i_corr_ estimates were obtained from R_p_ measurements, and ρ was estimated using a cell constant (C_C_) based on the dimensions of the specimen, giving the following formula:

ρ = C_C_·Rs (2)

where C_C_ = 7.4 is the cell constant obtained in a previous investigation [3]. Figure 9 presents the composite plots of ρ vs. the electrochemical parameter i_corr_ for all data obtained in this experimental program.

As observed from Figure 9, there is an excellent correlation (R^2^~0.93) between mortar´s electrical resistivity (ρ) and the corrosion rate (i_corr_) of the steel, regardless of mortar type (with or without OFI mucilage addition). There is no difference whatsoever between the mortar mixtures tested for the corrosion activity and ionic conductivity (inverse of the electrical resistivity), and all data follow a similar trend: higher values of ρ produce lower values of i_corr_, as observed from a previous investigation on carbonated concrete [16].

**Figure 10:**
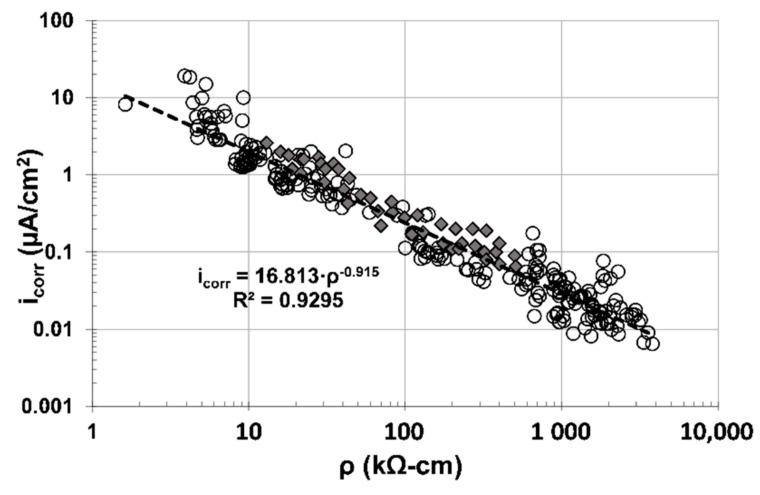
Composite plots of ρ and i_corr_ for all mortar treatments evaluated (○) and data from previous investigations [16] using concrete specimens (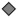).

Figure 10 shows results from this investigation as one cloud of data (○) and results from a previous investigation (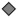) where concrete specimens were evaluated [16]. It also includes the empirical correlation equation and coefficient using both data clouds together. As observed in Figure 10, an excellent correlation between ρ and i_corr_ was obtained, and corroborates previous investigations that determined i_corr_ of reinforced concrete infrastructure by measuring ρ only [17,18].

As a final discussion, based on the information obtained in this and previous investigations, there is an apparent improvement of the durability of cement-based materials when OFI mucilage is used as an addition. As presented in a previous investigation [4], the addition of OFI mucilage in mortar decreases CO_2_ penetration into mortar. Therefore, OFI mucilage increased the time before corrosion initiation on steel in cement-based mortar in a CO_2_-laden environment.

The results on the cracking performance of all tested mortars obtained after 5110 days in a natural CO_2_-laden environment conclude that OFI mucilage also increased the time before corrosion propagation on steel in carbonated mortar. The performance was observed in a previous investigation [4] and, in this investigation, suggests that OFI mucilage could be used as a durability enhancer for steel in cement-based materials in both corrosion stages: initiation and propagation.

## 5. Conclusions

The experimental findings suggest that adding Opuntia ficus-ndica (OFI) mucilage might be useful as a corrosion inhibitor for steel in carbonated cement-based materials (i.e., mortar):

1. Electrochemical results demonstrated the durability enhancement of OFI mucilage additions in carbonated mortar, because corrosion rates of the embedded steel decreased when mortar had enough water in its pores to promote corrosion.
2. The inhibitor efficiencies obtained with OFI mucilage additions, in carbonated mortar, were 40%–70% for low OFI mucilage concentrations (1.5% and 4%), and 70–90% for medium/high OFI mucilage concentrations (8%, 42%, and 95%).
3. Experimental results also demonstrated that corrosion-induced surface cracking was diminished in carbonated mortar with OFI mucilage additions.
4. Based on the previous conclusions, OFI mucilage added to mortar increased the corrosion propagation period of the embedded steel, because corrosion rates were decreased and cracking initiation and propagation were diminished.
5. An excellent empirical correlation was observed for the electrochemical parameters corrosion potential (E_corr_) and corrosion rate (i_corr_), with a ~−110 mV/decade slope.
6. Another empirical correlation was determined between the mortar electrical resistivity (ρ) and the steel bars’ i_corr_, giving an almost linear dependence between these two parameters: i_corr_ = 16.8·ρ^−0.915^ with R^2^ = 0.93. This correlation was corroborated by experimental data obtained from this and previous investigations, thus, an application to obtain the corrosion rate of steel embedded in cement-based materials only by measuring the electrical resistivity of the mortar/concrete cover is promising.

## Figures and Tables

**Table 1:** Mixture proportions and mortar flow values for each mixture tested [3,4].

Mortar Mixture	w/c or (w + m)/c Ratio	Cement (kg/m^3^)	Water (kg/m^3^)	Mucilage (kg/m^3^)	Sand (kg/m^3^)	Flow (%)
CPO	0.88	307.4	270.0	0	845.2	113.4
1.5% B	0.88	307.4	264.8	5.2	845.2	113.0
4% B	0.88	307.4	259.6	10.4	845.2	113.8
8% B	0.88	307.4	249.2	20.8	845.2	114.6
42% B	0.88	307.4	166.1	103.9	845.2	116.3
95% B	0.88	307.4	10.4	259.6	845.2	128.9

## Data Availability

The data presented in this study are available on request from the corresponding author.

## References

[1] Hernández E.F., Cano-Barrita P.F.D.J., León-Martínez F.M., Torres-Acosta A.A. (2017). Performance of cactus mucilage and brown seaweed extract as a steel corrosion inhibitor in chloride contaminated alkaline media. Anti-Corrosion Methods Mater..

[2] Hernández E.F., Cano-Barrita P.F.J., Torres-Acosta A.A. (2016). Influence of cactus mucilage and marine brown algae extract on compressive strength and durability of concrete. Mater. Constr..

[3] Martinez-Molina W., Torres-Acosta A.A., Hernández-Leos R., Alonso Guzmán E., Mendoza-Pérez I.N., Martínez-Peña G.E.I. (2016). The Inhibitive Properties of Nopal Slime on the Corrosion of Steel in Chloride-Contaminated Mortar. Anti-Corros. Methods Mater..

[4] Torres-Acosta A.A., González-Calderón P.Y. (2020). Mortar with OFI mucilage additions exposed to CO_2_-laden environment. ACI Mate. J..

[5] Martinez-Molina W., Torres-Acosta A.A., Martínez-Peña G.E.I., Guzmán E.A., Mendoza-Pérez I.N. (2015). Cement-Based Materials Enhanced Durability from Opuntia Ficus Indica Mucilage Additions. ACI Mater. J..

[6] Torres-Acosta A.A., Díaz-Cruz L.A. (2020). Concrete durability enhancement from nopal (opuntia ficus-indica) additions. Constr. Build. Mater..

[7] Martinez-Molina W., Torres-Acosta A.A., Celis-Mendoza C.E., Alonso-Guzmán E. (2014). Physical Properties of Cement-Based Paste and Mortar With Dehydrated Cacti Additions. Int. J. Arch. Heritage.

[8] Torres-Acosta A.A. (2019). Water and chloride permeability of cement-based mortar with additions of dehydrated cacti. J. Chem. Technol. Biotechnol..

[9] Torres-Acosta A.A. (2007). Opuntia-Ficus-Indica (Nopal) mucilage as a steel corrosion inhibitor in alkaline media. J. Appl. Electrochem..

[10] Torres-Acosta A.A., Martínez-Madrid M., Loveday D.C., Horner M. Nopal and Aloe Vera Additions in Concrete: Electrochemical Behavior of the Reinforcing Steel. Proceedings of the CORROSION 2005.

[11] Sáenz C., Sepúlveda E., Matsuhiro B. (2004). Opuntia spp mucilage’s: A functional component with industrial perspectives. J. Arid. Environ..

[12] ONNCEE Standard (1999). Building Industry–Hydraulic Cement–Specifications and Testing Methods.

[13] American Society for Testing and Materials (2003). Standard Specification for Flow Table for Use in Tests of Hydraulic Cement.

[14] American Society for Testing and Materials (2006). Standard Practice for Mechanical Mixing of Hydraulic Cement Pastes and Mortars of Plastic Consistency.

[15] Sagüés A., Pech-Canul M., Al-Mansur A.S. (2003). Corrosion macrocell behavior of reinforcing steel in partially submerged concrete columns. Corros. Sci..

[16] Geiker M., Hornsbostel K., Belda Revert A. Corrosion rate; experimental observations in carbonated or chloride containing concrete. Proceedings of the Mathematical Models of Reinforcement Corrosion and Its Consequences for Structural Performance.

[17] Alonso C., Andrade C., González J.A. (1988). Relation between resistivity and corrosion rate of reinforcements in carbonated mortar made with several cement types. Cem. Concr. Res..

[18] Feliu S., Andrade C., González J.A., Alonso C. (1996). A new method forin-situ measurement of electrical resistivity of reinforced concrete. Mater. Struct..

